# Gelatine Treatment in Aneurysm

**Published:** 1903-10

**Authors:** Ennion C. Williams

**Affiliations:** Richmond, Va., Professor of Pathology Medical College of Virginia, Pathologist to the Memorial Hospital, etc.


### GELATINE TREATMENT IN ANEURYSM.*

BY ENNION C. WILLIAMS, M.D., RICHMOND, VA.,

Professor of Pathology Medical College of Virginia, Pathologist to the Memorial Hospital, etc.

In 1901 I made a preliminary report of a case of abdominal aneurysm in a colored woman forty years of age, treated with injections of a solution of gelatine. The mass extended from the ensiform cartilage to within five centimeters of the umbilicus, and laterly to the parasternal line on the right side and slightly beyond the parasternal line on the left side, producing a markedly visible elevation. It was rather firm and expansile. A loud bruit could be heard over it.

The patient was very emaciated and had been confined to her bed most of the time during the preceding year, and constantly for the

*Read before the Richmond Academy of Medicine and Surgery, July 28, 1903.

previous six weeks. The pain was so severe that morphine had to be taken regularly every day and night.

The first injection of gelatine, on September 16, consisted of 10 c.c. of a warmed aqueous solution containing 10 per cent. gelatine and 2 per cent. calcium chloride. On September 29 a similar injection was given. On October 7, 15 c.c. of a solution containing 10 per cent. gelatine only was given; October 15, 20 c.c.; October 21, 15 c.c. and November 4, 20 c.c. of a solution containing 5 per cent. of gelatine. This made a total in seven weeks of six injections—90 c.c. of solution containing 6½ grams of gelatine and 4-10 gram of calcium chloride.

At the end of this seven weeks' treatment the patient said she had not felt so well for more than a year. There was no longer the continuous pain in the back and sides. She took morphine only occasionally at night, and was so much stronger that she spent much of her time out of bed, and could walk about with comparative ease. The aneurysmal mass was firmer and the bruit less distinct and of a higher pitch. The injections of gelatine solution were continued through the following eight months at intervals of about two weeks, but larger injections were given, from 20 c.c. to 60 c.c. of the solution at each injection. The patient continued to grow stronger and could walk up and down the stairs and out in the street. She never gave up the morphine entirely, but would usually take ¼ of a grain at night. Towards the following summer the pains increased and her strength grew less, although the abdominal mass was apparently the same. The injections did not give her the same relief. In July, after two weeks of agonizing pain in the left side which morphine would not relieve, she died.

On account of my absence from the city Dr. Greer Baughman kindly held the autopsy for me, three hours after death. The arteries were sclerotic; the abdominal cavity was filled with clots. The aneursymal mass was adherent to the liver, stomach and intestines, and partly to the diaphragm. The adhesions were so dense and the organs so massed together that great difficulty was experienced in separating them. The dilatation extended from two inches of the iliac bifurcation to and including part of the thoracic aorta. The 7th, 8th and 9th dorsal vertebræ were eroded to a considerable extent. The large sac of the aneurysm was anterior. Against the anterior wall of this sac, at the summit, there was a dense laminated ante-mortem coagulum, crescent-shaped and thickest in the middle. This coagulum did not appear to be very closely adherent to the wall, as there seemed to be a very narrow space (1 mm.) between the wall and the clot. Microscopic examination of the clot showed it to consist of a dense mass of fibrin apparently deposited in layers at different times. Within the cavity of the aneurysm was much coagulated blood, more than one would expect only three hours post mortem. This firm, dense clot lining the anterior part of the sac of the aneurysm readily accounted for the relief of pain and throbbing at first experienced by the patient, and the tumor becoming firmer and less pulsating. Posteriorly, the aneurysm had eroded three vertebræ, leaving not much more than the upper and lower surfaces and the intervening cartilages. The rupture was found to have taken place posteriorly. The erosion accounted for the pain never having been entirely relieved and increasing so greatly toward the close.

Another case was that of a colored man, aged fifty-five: a hard drinker, with sclerotic arteries. He had had pain in his chest for two years. There was a large, pulsating expansile mass projecting just to the right of the sternum. At intervals of about ten days he was given three injections, 30 to 50 c.c., of a solution containing ten per cent. gelatine. He said that several days after the injections the pain was much less. He left the hospital intending to return, but grew worse, and a month later died.

A post mortem was made five hours after death. No rupture was found in the aneurysm. The dilatation began at the aortic orifice and extended to the descending aorta. There were some clots in the aneurysm, but they were all doubtless post mortem. The third, fourth and fifth ribs were eroded through, and the second and sixth partially eroded. The right lung was collapsed, and the left emphysematous. The abdominal and pleural cavities were filled with yellow fluid. The feet and legs were greatly swollen and edematous.

I have given the injection to two other cases of suspected aneurysm of the thoracic aorta, but without result except that the patients believed that the pain was temporarily relieved.

In the treatment of the first case mentioned, from two to three hours after the first few injections the patient had a chill followed by an elevation of temperature, which, when I saw her the next morning, was 100° to 101°. This gradually subsided in twenty-four hours. Although the quantity of gelatine was kept up, the reaction lessened until there was neither chill nor fever. The lessening of the reaction was partly coincident with the use of a purer gelatine rather than the gelatine bought in bulk for making culture media. In the second case, in which the purer gelatine was used, there was no chill and only a very slight rise in temperature. This was the result in the other two cases.

Just what effect the calcium chloride has I have not sufficient experience to say. It was added because it has the reputation of increasing the coagulability of the blood. I thought it hindered the prompt absorption of the solution, and, therefore, left it out after the first two injections.

During the treatment of the first case I made fifty-one tests of the coagulative power of the blood, with a modification of Wright's Coagulamater, before and after the injections. I will only summarize the results. Before an injection of 50 c.c. of a 10 per cent. solution of gelatine the blood coagulated in two and a half minutes; thirty minutes were consumed in giving the injection. Five minutes after the last injection the blood coagulated in one and a half minutes. Twenty-four hours after another injection the blood coagulated in one and a half minutes, having coagulated before the injection in two and a half minutes. Three days afterwards it coagulated in one and a half minutes, and five days afterwards in two minutes. These tests tend to show that the gelatine promptly increases the coagulative power of the blood, and that the blood retains this power at least five days.

The solution used for the injections is prepared by adding the gelatine gradually to water just below the boiling point, and keeping the solution at a slow boil for ten minutes. It is then filtered and kept in small flasks plugged with sterile cotton. It should be sterilized in a steam sterilizer for thirty minutes on three successive days.

The deaths so far reported in this treatment have been due to the gelatine being contaminated with tetanus germs. Great care should, therefore, be used to thoroughly sterilize the solution. At the same time it should be noted that excessive heating weakens the coagulative power of the gelatine. Just before administering, the solution must be thoroughly liquefied by immersing the flask in hot water. It is needless to mention the usual aseptic precautions necessary in using the antitoxin syringe.

My experience has been that there is hardly any pain if the injection is given carefully and slowly, and with the solution hardly above the temperature of the body. I have given the injections beneath the breasts, in the back and in the thighs. In the selection of the site one can consult their convenience. I usually apply a hot-water bag after the injection to hasten the absorption. The patient should be kept absolutely quiet for at least five days, and then allowed to move about a little until the next injection, two to ten days later, to keep up the general health.

I believe the blood will only clot in saccular aneurysms, and the treatment is applicable only to such cases.

Lancereux, who first announced this treatment, and others who have since reported cases, have used 1 per cent. to 2 per cent. gelatine in normal salt solution, and given 200 to 250 c.c. at one injection. I prefer to use a 5 to 10 per cent. solution, and to give about 50 c.c. at one injection instead of 200 c.c. of a 1 to 2 per cent. solution; because the larger the quantity of solution injected hypodermically, the greater is the circulation stimulated. If the same amount of gelatine can be injected with a smaller quantity of solution, the greater will be the tendency of the blood to clot.

In conclusion, the treatment is one that should be given a thorough trial. So far it seems to be harmless if the solution is properly sterilized. It is practically painless if given with due care. The gelatine undoubtedly increases the coagulative power of the blood. In the treatment of aneurysm, it is probably of value only in cases of the saccular type. It is doubtless of great value in many cases of hemorrhage and other conditions where a prompt increase in the coagulative power of the blood is desired.

315 Grace Street, East.

